# Lactate dehydrogenase: relationship with the diagnostic GLIM criterion for cachexia in patients with advanced cancer

**DOI:** 10.1038/s41416-022-02099-5

**Published:** 2022-12-14

**Authors:** Josh McGovern, Ross D. Dolan, Claribel P. L. Simmons, Louise E. Daly, Aoife M. Ryan, Derek G. Power, Donogh Maguire, Marie T. Fallon, Barry J. Laird, Donald C. McMillan

**Affiliations:** 1 Academic Unit of Surgery, School of Medicine, University of Glasgow, New Lister Building, Glasgow Royal Infirmary, Glasgow, Scotland; 2 Institute of Genetics and Molecular Medicine, University of Edinburgh, Edinburgh, Scotland; 3 School of Food and Nutritional Sciences, College of Science, Engineering and Food Science, University College Cork, Cork, Ireland; 4 Department of Medical Oncology, Mercy and Cork University Hospital, Cork, Ireland

**Keywords:** Cancer metabolism, Glycobiology

## Abstract

**Background:**

Although suggestive of dysregulated metabolism, the relationship between serum LDH level, phenotypic/aetiologic diagnostic Global Leadership Initiative on Malnutrition (GLIM) criteria and survival in patients with advanced cancer has yet to examined.

**Methods:**

Prospectively collected data from patients with advanced cancer, undergoing anti-cancer therapy with palliative intent, across nine sites in the UK and Ireland between 2011–2016, was retrospectively analysed. LDH values were grouped as <250/250–500/>500 Units/L. Relationships were examined using χ^2^ test for linear-by-linear association and binary logistics regression analysis.

**Results:**

A total of 436 patients met the inclusion criteria. 46% (*n* = 200) were male and 59% (*n* = 259) were ≥65 years of age. The median serum LDH was 394 Units/L and 33.5% (*n* = 146) had an LDH > 500 Units/L. LDH was significantly associated with ECOG-PS (*p* < 0.001), NLR (*p* < 0.05), mGPS (*p* < 0.05) and 3-month survival (*p* < 0.001). LDH was significantly associated with 3-month survival independent of weight loss (*p* < 0.01), BMI (*p* < 0.05), skeletal muscle mass (*p* < 0.01), metastatic disease (*p* < 0.05), NLR (*p* < 0.05) and mGPS (*p* < 0.01).

**Discussion:**

LDH was associated with performance status, systemic inflammation and survival in patients with advanced cancer. LDH measurement may be considered as an aetiologic criteria and become a potential therapeutic target in the treatment of cancer cachexia.

## Introduction

Present in almost every tissue in the human body, lactate dehydrogenase (LDH) is found in high concentrations in the liver, kidneys, and muscle [1]. In addition to acting as a functional checkpoint for glucose restoration during gluconeogenesis and single-stranded DNA metabolism, LDH is a key enzyme in anaerobic cell metabolism [2], converting lactate to pyruvate in the liver, via the Cori cycle [3]. Furthermore, LDH is also released by cells following damage to tissues, with a detectable rise in serum concentration levels observed [1].

Elevated serum LDH levels have also been reported to be associated with disease progression and metastasis in patients cancer [4] and has been shown to have prognostic value in relation to treatment efficacy [5, 6] and survival [7, 8]. The basis of such an association is thought to be the result of a combination of tumour necrosis due to hypoxia and enhanced glycolytic activity of the tumour (Warburg effect). As such, the role of LDH in cancer remains an area of interest and a potential therapeutic target in oncology [9, 10].

Although clearly of metabolic origin and having prognostic value in patients with advanced cancer [11], where cachexia is prevalent [12], circulating LDH level has rarely been examined in patients with cancer cachexia. Specifically, the relationship with the phenotypic and aetiologic criterion utilised in the current global consensus on the diagnosis of cancer cachexia- the Global Leadership Initiative on Malnutrition (GLIM) criteria. Therefore, the aim of the present study was to examine the relationship between serum LDH level, diagnostic GLIM criterion and survival in patients with advanced cancer.

## Methods

### Patients

Prospectively collected data from patients with advanced cancer, undergoing anti-cancer therapy with palliative intent, across nine sites in the UK and Ireland between 2011–2016, was retrospectively analysed [13, 14]. Eligible adult patients with advanced disease (defined as locally advanced or with histological, cytological or radiological evidence of metastasis), across all cancer subtypes, who had recorded serum LDH values prior to entry to the study were assessed for inclusion. The study included patients with primary lung, gastro-intestinal, breast, gynaecological, urological and haematological malignancies. The study had ethical approval in both the UK and Ireland (West of Scotland Ethics Committee UK: 18/WS/0001 (18/01/2018) and Cork Research Ethics Committee Ireland: ECM 4 (g) (03/03/2015) and was conducted in accordance with the Declaration of Helsinki, as previously described [13, 14]. The study conformed to the Strengthening the Reporting of Observational Studies in Epidemiology (STROBE) guidelines for cohort studies [15].

General demographic data and clinicopathological characteristics were recorded for each patient prior to study entry. Tumour site was grouped as lung, gastrointestinal (GI) or other. Eastern Co-operative Oncology Group Performance Status (ECOG-PS) was determined by a clinician or clinical researcher at the institute the patient was receiving treatment. Patients were categorised according to their ECOG-PS into five grades (grade 0–4) and then grouped as 0-1/2/3-4, as previously described [16]. Serum lactate dehydrogenase (LDH) were calculated from venous blood values. LDH values were grouped as <250/250–500/>500 Units/L based on threshold values in the literature [7]. The primary outcome of interest was survival three months from entry to the study.

### GLIM criterion for diagnosing cancer cachexia

As proposed by Cederholm and co-workers international criteria consensus, a diagnosis of cancer cachexia requires the presence of one of three phenotypic (involuntary weight loss, low BMI, low muscle mass) and one tumour aetiologic criteria (reduced food intake or assimilation and inflammation/disease burden [17]. Each patient had weight and BMI recorded on entry to the study. Weight loss was categorised as (≤/>5%) prior to study entry. A low BMI as <20 kg/m^2^ in patients aged <70 years and <22 kg/m^2^ in patients aged >70 years. A low skeletal muscle mass was defined as a low SMI calculated from CT-images at the level of the third lumbar vertebra, as described below. Disease burden was classified as the presence/absence of metastasis on staging CT scan prior to entry to the study. Presence of inflammation was determined using the neutrophil/lymphocyte ratio (NLR) and the modified Glasgow Prognostic Score (mGPS), calculated from venous blood samples obtained on entry to the study. The NLR was calculated by division of the neutrophil count by the lymphocyte count, obtained from the patient’s full blood count (FBC) and values were grouped as <3/3–5/>5 [18]. The mGPS was calculated as previously described and grouped as 0/1/2 [19]. An autoanalyzer was used to measure serum CRP (mg/L) and albumin (g/L) concentrations according to routine clinical laboratory protocols.

### CT-derived skeletal muscle mass

CT images were obtained at the level of the third lumbar vertebra as previously described [20]. Patient scans were taken within three months prior to study entry. Scans with significant movement artefact or missing region of interest were not considered for inclusion. Each image was analysed using a free-ware program (NIH Image J version 1.47, http://rsbweb.nih.gov/ij/) shown to provide reliable measurements [21].

Region of interest measurement was made of the skeletal muscle area (SMA) (cm^2^) using standard Hounsfield Unit (HU) range (−29 to +150 HU). These were then normalised for height^2^ to create the skeletal muscle index (SMI, cm^2^/m^2^). A low SMI was defined as described by Martin and colleagues and an SMI < 43cm^2^/m^2^ if BMI < 25 kg/m^2^ and SMI < 53cm^2^/m^2^ if BMI ≥ 25 kg/m^2^ in male patients and SMI < 41cm^2^/m^2^ in female patients if BMI < or ≥25 kg/m^2^ [22].

### Statistical analysis

Demographic data, clinicopathological variables, LDH, ECOG-PS, weight loss, BMI, SMI, NLR, mGPS and 3-month survival were presented as categorical variables. Categorical variables were analysed using χ^2^ test for linear-by-linear association.

Demographic data, clinicopathological variables, LDH, ECOG-PS, weight loss, BMI, SMI, NLR, mGPS and 3-month survival were examined using univariate and multivariate binary logistic regression, to calculate Odds ratios and 95% Confidence Intervals. Clinicopathological factors that had a *p* value <0.1 were taken into a multivariate model using a backward conditional model to identify independently significant factors.

Missing data were excluded from analysis on a variable-by-variable basis. Two-tailed *p* values <0.05 were considered statistically significant. Statistical analysis was performed using SPSS software version 25.0. (SPSS Inc., Chicago, IL, USA).

## Results

### Patient Inclusion

**Fig. 1:**
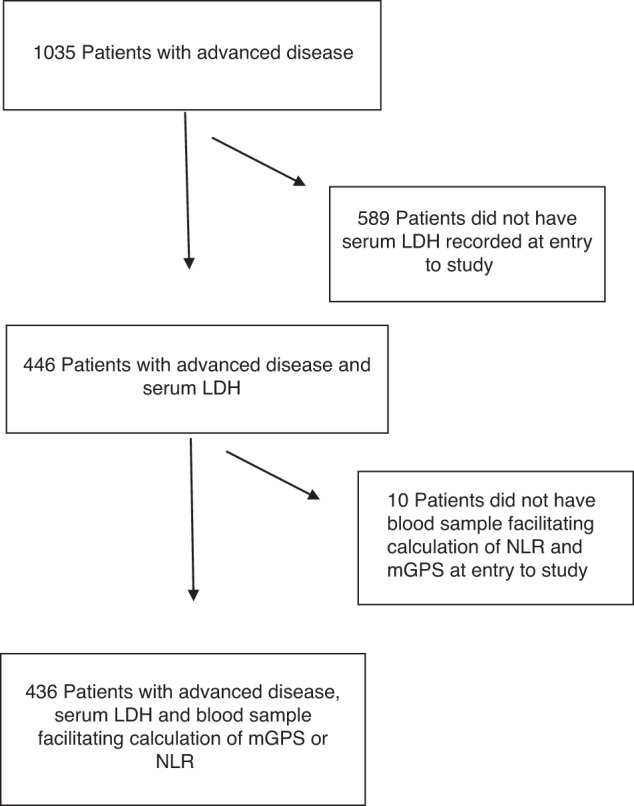
Flow diagram of included patients. LDH Lactate dehydrogenase, NLR Neutrophil: lymphocyte ratio, mGPS modified Glasgow Prognostic Score.

A total of 436 patients met the inclusion criteria (see Fig. 1). The clinicopathological characteristics of the included patients are shown in Table 1. 46% (*n* = 200) were male and 59% (*n* = 259) were ≥65 years of age. The majority of patients had either lung (37%, *n* = 162) or GI (28%, *n* = 124) tumours. 61% (*n* = 267) of patients received chemotherapy, 41% (*n* = 179) received radiotherapy and 14% (*n* = 59) received hormonal therapy. The median serum LDH was 394 Units/L (1.8–2757) and 34% (*n* = 146) had an LDH > 500 Units/L. 41% (*n* = 180) of patients were ECOG-PS 0/1. Of the 421 patients, 33% (*n* = 139) had >5% weight loss. 33% (*n* = 143) patients were categorised as having a low BMI. Of the 177 patients with CT-imaging facilitating body composition analysis, 55% (*n* = 97) were categorised as having a low skeletal muscle mass. 81% (*n* = 355) patients had metastatic disease on entry to the study. 44% (*n* = 193) patients had an NLR > 5 and 62% (*n* = 270) patients had an mGPS ≥ 1. The median survival from study entry was 8.7 months (0–22) and 65% (*n* = 284) of patients were alive at 3-months from entry to the study.

**Table 1:** The relationship between serum LDH, clinicopathological variables, weight loss, BMI, skeletal muscle mass, disease burden, systemic inflammation and survival in patients with advanced cancer, stratified by LDH (*n* = 436).

	LDH < 250 Units/L (*n* = 110)	LDH 250–500 Units/L (*n* = 180)	LDH > 500 Units/L (*n* = 146)	*P* value^a^
Age				0.101
<65	48 (44)	72 (40)	57 (39)	
65–74	38 (35)	50 (28)	39 (26)	
>74	23 (21)	58 (32)	50 (34)	
Sex				0.412
Female	52 (47)	106 (58)	78 (53)	
Male	58 (53)	74 (41)	68 (47)	
Tumour site				0.266
Lung	51 (46)	66 (37)	45 (31)	
GI	20 (18)	50 (28)	54 (37)	
Other	39 (36)	64 (35)	47 (32)	
Chemotherapy				0.248
Yes	61 (56)	114 (63)	92 (63)	
No	49 (44)	66 (37)	54 (37)	
Radiotherapy				0.427
Yes	39 (35)	80 (44)	60 (41)	
No	71 (65)	100 (56)	86 (59)	
Hormone therapy				0.136
Yes	13 (12)	20 (11)	26 (18)	
No	97 (88)	160 (89)	120 (82)	
ECOG-PS				<0.001
0/1	69 (63)	68 (38)	43 (30)	
2	30 (27)	87 (48)	62 (42)	
3/4	11 (10)	25 (14)	41 (28)	
Weight loss (>5%)^b^				0.662
No	70 (65)	116 (67)	96 (68)	
Yes	37 (35)	57 (33)	45 (32)	
Low BMI				0.273
No	84 (76)	109 (61)	100 (68)	
Yes	26 (24)	71 (39)	46 (32)	
Low skeletal muscle mass^c^				0.210
No	33 (50)	25 (46)	22 (39)	
Yes	33 (50)	29 (54)	35 (61)	
Metastatic disease				0.118
No	20 (18)	44 (24)	17 (12)	
Yes	90 (82)	136 (76)	129 (88)	
NLR				0.003
<3	43 (39)	64 (35)	44 (30)	
3–5	31 (28)	41 (23)	20 (14)	
>5	36 (33)	75 (42)	82 (56)	
mGPS				0.021
0	50 (45)	77 (43)	39 (27)	
1	14 (13)	38 (21)	38 (26)	
2	46 (42)	65 (36)	69 (47)	
3-month survival				<0.001
Yes	93 (93)	121 (67)	70 (47)	
No	17 (17)	59 (33)	76 (53)	

^a^*p* value is from χ2 analysis.

^b^15 patients did not have sequential monitoring of weight.

^c^249 patients did not have eligible CT imaging at L3 for CT-body composition analysis.

The relationship between LDH and ECOG-PS, weight loss, BMI, SMI, NLR, mGPS and survival in patients with advanced cancer is shown in Table 1. LDH was significantly associated with ECOG-PS (*p* < 0.001), NLR (*p* < 0.05), mGPS (*p* < 0.05) and 3-month survival rate (*p* < 0.001). It was not associated with age (*p* = 0.101), sex (*p* = 0.412), tumour site (*p* = 0.266), chemotherapy (*p* = 0.248), radiotherapy (*p* = 0.427), hormone therapy (*p* = 0.136), weight loss (*p* = 0.662), low BMI (*p* = 0.273), low skeletal muscle mass (*p* = 0.210) or metastatic disease (*p* = 0.118).

The relationship between LDH, weight loss and 3-month survival in patients is shown in Table 2a. LDH was significantly associated with 3-month survival independent of weight loss (*p* < 0.01). The relationship between LDH, BMI and 3-month survival in patients with advanced cancer is shown in Table 2b. LDH was significantly associated with 3-month survival independent of BMI (*p* < 0.05). The relationship between LDH, SMI and 3-month survival in patients with advanced cancer is shown in Table 2c. LDH was significantly associated with 3-month survival independent of SMI (*p* < 0.01). The relationship between LDH, disease burden and 3-month survival in patients with advanced cancer is shown in Table 2d. LDH was significantly associated with 3-month survival independent of the presence of metastatic disease (*p* < 0.05). The relationship between LDH, NLR and 3-month survival in patients with advanced cancer is shown in Table 2e. LDH was significantly associated with 3-month survival independent of NLR > 5 (*p* < 0.05). The relationship between LDH, mGPS and 3-month survival in patients with advanced cancer is shown in Table 2f. LDH was significantly associated with 3-month survival independent of mGPS (*p* < 0.01).

**Table 2:** a. The relationship between serum LDH, weight loss and 3-month survival in patients with advanced cancer (*n* = 421). b. The relationship between serum LDH, low BMI and 3-month survival in patients with advanced cancer (*n* = 436). c. The relationship between serum LDH, low skeletal muscle mass and 3-month survival in patients with advanced cancer (*n* = 177). d. The relationship between serum LDH, disease burden and 3-month survival in patients with advanced cancer (*n* = 436). e. The relationship between serum LDH, NLR and 3-month survival in patients with advanced cancer (*n* = 436). f. The relationship between serum LDH, mGPS and 3-month survival in patients with advanced cancer (*n* = 436).

a				
	LDH < 250 Units/L (*n* = 107)	LDH 250–500 Units/L (*n* = 173)	LDH > 500 Units/L (*n* = 141)	*P* value^a^
Weight loss ≤ 5% (*n* = 282)	63 (57%)	92 (51%)	51 (35%)	<0.001
Weight loss > 5% (*n* = 139)	28 (25%)	26 (14%)	18 (12%)	0.002
*P* value^a^	0.048	<0.001	0.146	
b				
	LDH ≤ 250 Units/L (*n* = 110)	LDH 250–500 Units/L (*n* = 180)	LDH > 500 Units/L (*n* = 146)	*P* value^a^
Normal/high BMI (n = 293)	78 (73%)	82 (47%)	54 (38%)	<0.001
Low BMI (*n* = 143)	17 (16%)	39 (23%)	16 (11%)	0.008
*P* value^a^	0.002	0.005	0.031	
c				
	LDH ≤ 250 Units/L (*n* = 66)	LDH 250–500 Units/L (*n* = 54)	LDH > 500 Units/L (*n* = 57)	*P* value^a^
Normal/high SMI (*n* = 80)	31 (47%)	18 (33%)	14 (25%)	0.006
Low SMI (*n* = 97)	28 (42%)	22 (41%)	17 (30%)	0.001
*P* value^a^	0.230	0.747	0.266	
d				
	LDH ≤ 250 Units/L (*n* = 110)	LDH 250–500 Units/L (*n* = 180)	LDH > 500 Units/L (*n* = 146)	*P* value^a^
Non-metastatic disease (*n* = 81)	17 (15%)	31 (17%)	9 (6%)	0.035
Metastatic disease (*n* = 355)	76 (69%)	90 (50%)	61 (42%)	<0.001
*P* value^a^	0.950	0.599	0.661	
e				
	LDH < 250 Units/L (*n* = 110)	LDH 250–500 Units/L (*n* = 180)	LDH > 500 Units/L (*n* = 146)	*P* value^a^
NLR < 3 (*n* = 151)	41 (37%)	57 (32%)	31 (21%)	0.001
NLR 3–5 (*n* = 92)	28 (25%)	25 (14%)	14 (10%)	0.057
NLR > 5 (*n* = 193)	24 (22%)	39 (22%)	25 (17%)	<0.001
*P* value^a^	0.001	<0.001	<0.001	
f				
	LDH < 250 Units/L (*n* = 110)	LDH 250–500 Units/L (*n* = 180)	LDH > 500 Units/L (*n* = 146)	*P* value^a^
mGPS 0 (*n* = 166)	49 (45%)	67 (37%)	30 (21%)	0.002
mGPS 1 (*n* = 90)	12 (11%)	25 (14%)	17 (12%)	0.005
mGPS 2 (*n* = 180)	32 (29%)	29 (16%)	23 (16%)	<0.001
*P* value^a^	<0.001	<0.001	<0.001	

Each cell (*n* = /%).

^a^*p* value is from χ2 analysis.

The relationship between clinicopathological variables, ECOG-PS, weight loss, low BMI, NLR, mGPS, LDH and 3-month survival in patients with advanced cancer is shown in Table 3. On univariate analysis, chemotherapy (*p* < 0.001), ECOG-PS (*p* < 0.001), weight loss (*p* < 0.05), low BMI (*p* < 0.001), NLR (*P* < 0.001), mGPS (*p* < 0.001) and LDH (*p* < 0.001) were significantly associated with 3-month survival. On multivariate analysis, chemotherapy (*p* < 0.05), ECOG-PS (*p* < 0.05), NLR (*P* < 0.001), mGPS (*p* < 0.001) and LDH (*p* < 0.05) remained significantly associated with 3-month survival.

**Table 3:** The relationship between clinicopathological variables, ECOG-PS, weight loss, low BMI, NLR, mGPS, serum LDH and 3-month survival in patients with advanced cancer (*n* = 436).

	Univariate analysis		Multivariate analysis	
	*Odds ratio (95% Confidence Interval)*	*p*-value	*Odds ratio (95% Confidence Interval)*	*p*-value
Age (<65/65–74/>74)	1.13 (0.90–1.43)	0.299	–	–
Sex (male/female)	0.91 (0.62–1.33)	0.616	–	–
Chemotherapy (yes/no)	0.44 (0.29–0.67)	<0.001	0.57 (0.35–0.92)	0.022
ECOG-PS (0–1/2/≥3)	2.69 (1.99–3.64)	<0.001	1.73 (1.22–2.45)	0.002
Weight loss (≤5/>5%)	1.93 (1.26–2.95)	0.003	–	0.365
Low BMI (yes/no)	2.36 (1.53–3.62)	<0.001	–	0.109
NLR (<3/3–5/>5)	2.29 (1.82–2.90)	<0.001	1.70 (1.30–2.22)	<0.001
mGPS (0/1/2)	1.57 (2.03–3.27)	<0.001	1.97 (1.51–2.60)	<0.001
LDH (<250/250–500/>500)	1.73 (1.33–2.24)	<0.001	1.43 (1.05–1.94)	0.022

## Discussion

To our knowledge the present study is one of the largest to date examining the relationship between LDH and other validated prognostic host factors (specifically the GLIM criteria) in patients with advanced cancer. Therefore, it was of interest that LDH was shown to be significantly associated with performance status, systemic inflammation and survival but not weight loss, low BMI or low SMI. Also, compared with weight loss, low BMI and low SMI, LDH had superior prognostic value. Given that elevated LDH values are an early marker of dysfunctional glucose metabolism, the present observations may represent the tip of tumour/ host metabolic iceberg with other profound metabolic changes. In particular, elevated LDH was associated with the systemic inflammatory response which is in turn recognised to have a catabolic effect on skeletal muscle in patients with cancer [23]. Therefore, the present results would suggest that elevated LDH values would be a useful addition to the GLIM criteria as an aetiologic factor.

The results of the present study are consistent with the observations of Zhou and co-workers, who reported that, in 359 patients with small cell lung cancer, elevated LDH was significantly associated with mGPS [23]. The basis of this relationship is not clear. However, it has been reported that increased tumour and bone marrow glucose uptake was associated with systemic inflammation in different tumour types [24]. Specifically, at the tumour microenvironment level, inhibitors of LDH appear to reverse inflammation induced changes [25, 26]. Taken together, these observations appear to confirm intimate cellular connection between inflammation and metabolism as proposed by Hotamisligil and co-workers occur not only at the cellular, but also at the level of the whole body [27]. Therefore, it may be that the immune-metabolic changes that occur in the tumour microenvironment result in systemic increases in lactate and inflammation which the subsequently impact on skeletal muscle and performance status. This hypothesis requires testing both in the tumour microenvironment and at the systemic level in patients with cancer. Irrespective, the measurement of LDH and systemic inflammation in routine clinical cancer care would alert the clinician to the presence of profound immune-metabolic changes in the patients and the increased likelihood of poor survival.

There are a number of limitations to the present study. Firstly, this study is retrospective in nature and subject to sample bias. Indeed, less than half (42%, *n* = 177) of the included patients had eligible CT-imaging available for body composition analysis. Nevertheless, these routine available clinical results may be readily tested in future studies.

If the present results are confirmed in subsequent studies, then within the GLIM criteria, LDH measurement should be considered as an aetiologic criterion. In due course it may become a therapeutic target in the treatment of cachexia in patients with advanced cancer. In conclusion, serum LDH was associated with performance status, systemic inflammation and survival but with not weight loss, BMI or SMI in patients with advanced cancer.

## Supplementary information

Checklist

## Data Availability

Raw data will be made available on request to the senior author (BJL).
